# Supplementary material for: The differential burden of acute rhinovirus infections in children with underlying conditions
Source: PLoS One. 2025 May 14;20(5):e0313237. doi: 10.1371/journal.pone.0313237 (PMC12077780; doi:10.1371/journal.pone.0313237)
Supplement: S1 File — (DOCX) [file pone.0313237.s001.docx]

Supplementary Material

**The Differential Burden of Acute Rhinovirus Infections in Children with Underlying Conditions**

María Isabel Sánchez Códez^1^, Isabel Benavente Fernández^2,3,4^, Katherine Moyer^5^, Amy L. Leber^6^, Octavio Ramilo^7^, Asuncion Mejias^7^

# ^1^Division of Pediatric Infectious Diseases, Puerta del Mar, Cadiz, Spain; ^2^Department of Paediatrics, Puerta del Mar University Hospital, Cadiz, Spain; ^3^Biomedical Research and Innovation Institute of Cádiz (INiBICA) Research Unit, Spain; ^4^Area of Paediatrics, Department of Child and Mother Health and Radiology, Medical School, University of Cadiz, Spain; ^5^Division of Pediatric Infectious Diseases, Inova Children’s Hospital, Falls Church, VA,USA; ^6^ Department of Laboratory Medicine, Nationwide Children’s Hospital, Columbus, OH, USA; ^7^ Department of Infectious Diseases, St Jude Children’s Research Hospital, Memphis, TN, USA.

**S1 Table. Distribution of comorbidities in patients with underlying conditions** (n=1477).

| **Comorbidities** | **n (%)** |
| --- | --- |
| Asthma/atopy | 699 (47.3) |
| Chronic respiratory conditions **†** | 121 (8.2) |
| Prematurity | 147 (10.0) |
| CHD | 61 (4.2) |
| ICH | 27 (1.8) |
| Others |  |
| Genetic syndromes | 132 (8.9) |
| Gastrointestinal diseases | 68 (4.6) |
| Neurological disorders | 36 (2.4) |
| Hematologic disease | 17 (1.2) |
| Varied § | 169 (11.4) |

Data is presented as absolute numbers with percentages in parenthesis. **†**Chronic respiratory conditions included: cystic fibrosis (CF) (n=18); chronic lung disease (CLD) (n=61); tracheoesophageal fistula, pulmonary hypertension, spontaneous pneumothorax, airway malacia, subglottic stenosis, cleft palate, and Pierre–Robin sequence. §Endocrine, osteoarticular or genitourinary underlying diseases. CHD, congenital heart diseases; ICH, immunocompromised host.

**S2 Table. Distribution of underlying disease according to age.**

|  | **<1yr**  (n=934; 49.1%) | **1 to 5 yr**  (n=588; 31.0%) | **5 to 11 yr**  (n=246; 13.0%) | **11 to 21 yr**  (n=131; 6.9%) | **p value** |
| --- | --- | --- | --- | --- | --- |
| **Healthy** (n=422) | 279 (29.9)○ | 93 (15.8)○ | 31 (12.6)○ | 19 (14.5)○ | 0.007* |
| **Asthma/Atopy** (n=699) | 239 (25.6)○ | 264 (44.9)○ | 140 (56.9)○◻ | 56 (42.7)○◻ | 0.007* |
| **Chronic respiratory conditions †** (n=121) | 64 (6.8) | 33 (5.6) | 13 (5.3) | 11 (8.4) | >0.99 |
| **Prematurity** (n= 147) | 94 (10.1)○ | 45 (7.6)◻ | 6 (2.5)○◻ | 2 (1.5)○ | 0.007* |
| **CHD** (n=61) | 29 (3.1) | 18 (3.1) | 8 (3.2) | 6 (4.6) | >0.99 |
| **ICH** (n=27) | 2 (0.2)○ | 11 (1.9)○ | 8 (3.2)○ | 6 (4.6)○ | 0.007* |
| **Others §**(n=422) | 227 (24.3) | 124 (21.1) | 40 (16.3) | 31 (23.7) | 0.32 |

Data is presented as absolute numbers with percentages in parenthesis. CHD, congenital heart diseases; ICH, immunocompromised host. ***p*** values represent statistical significance between groups based on χ^2^ test. Asterisks (*****) indicate significant *p* values after applying the Bonferroni correction to adjust for multiple testing. Significant *p* values of <0.05 are represented by blank symbols (○, ◻). Matching symbols indicate the groups for which significant differences were observed.

**†** Chronic respiratory conditions included: cystic fibrosis (CF) (n=18); chronic lung disease (CLD) (n=61); tracheoesophageal fistula, pulmonary hypertension, spontaneous pneumothorax, airway malacia, subglottic stenosis, cleft palate, and Pierre–Robin sequence. **§** Others included: genetic syndromes (n=132); gastrointestinal diseases (n=68); neurological disorders (n=36); hematologic disease (n=17) and varied including endocrine, osteoarticular or genitourinary chronic comorbidities (n=169).

**S3 Table. Radiology findings in children and adolescents with RV infection.**

|  | **Previously healthy**  (n=422) | **Asthma/Atopy**  (n=699) | **Chronic respiratory conditions †** (n=121) | **Prematurity**  (n= 147) | **CHD**  (n=61) | **ICH**  (n=27) | **Others §**  (n=422) | **p value** |
| --- | --- | --- | --- | --- | --- | --- | --- | --- |
| **Radiology data** | | | | | | | |  |
| **Chest X-ray performed,** n (%) | 236 (55.9)○ | 541 (77.4)○◻ | 94 (77.7)○ | 111 (75.5)○ | 49 (80.3)○ | 16 (59.3) | 289 (68.5)○◻ | 0.0001* |
| **Chest X-ray abnormalities,** n (%) | 181/236 (76.7) | 442/541 (81.7)○ | 80/94 (85.1)◻ | 83/111 (74.8) | 41/49 (83.7) | 8/16 (50.0)○◻ | 233/289 (80.6) | 0.017* |
| **Bronchial wall thick/hyperinflat** | 63/236 (26.7)○ | 215/541 (39.7)○◻ | 30/94 (31.9) | 36/111 (32.4) | 13/49 (26.5) | 5/16 (31.2) | 87/289 (30.1)◻ | 0.0001* |
| **Atelectasis/Interstitial markings** | 69/236 (29.2) | 149/541 (27.5) | 32/94 (34.0) | 32/111 (28.8) | 16/49 (32.6) | 3/16 (18.7) | 84/289 (29.1) | 0.10 |
| **Lobar consolidation** | 37/236 (15.7) | 72/541 (13.3) | 16/94 (17.0) | 13/111 (11.7) | 10/49 (20.4) | 0 (0) | 49/289 (16.9) | 0.18 |
| **Other** | 12/236 (5.1) | 6/541 (1.1) | 2/94 (2.1) | 2/111 (1.8) | 2/49 (4.1) | 0 (0) | 13/289 (4.5) | 0.11 |

Data is presented as absolute numbers with percentages in parenthesis. CHD, congenital heart diseases; ICH, immunocompromised host. ***p*** values represent statistical significance between groups based on χ^2^ test. Asterisks (*****) indicate significant *p* values after applying the Bonferroni correction to adjust for multiple testing Significant *p* values of <0.05 are represented by blank symbols (○, ◻). Matching symbols indicate the groups for which significant differences were observed. **†** Chronic respiratory conditions included: cystic fibrosis (CF) (n=18); chronic lung disease (CLD) (n=61); tracheoesophageal fistula, pulmonary hypertension, spontaneous pneumothorax, airway malacia, subglottic stenosis, cleft palate, and Pierre–Robin sequence. **§** Others included: genetic syndromes (n=132); gastrointestinal diseases (n=68); neurological disorders (n=36); hematologic disease (n=17) and varied including endocrine, osteoarticular or genitourinary underlying diseases (n=169). Other Chest-X ray abnormalities include pulmonary edema, perihilar vascular congestion or calcifications.

|  | **Previously healthy**  (*n*=422) | **Asthma/Atopy**  (*n*=699) | **Chronic respiratory conditions †**  (*n*=121) | **Prematurity**  (*n*= 147) | **CHD**  (*n*=61) | **ICH**  (*n*=27) | **Others §**  (*n*=422) | ***p* value** |
| --- | --- | --- | --- | --- | --- | --- | --- | --- |
| **RV loads** (Ct values) | 25.6  [22.5-29.0] | 25.1  [21.9-29.0] | 24.1  [21.5-29.3] | 24.6  [21.5-28.2] | 24.3  [21.2-28.2] | 24.9  [22.8-28.6] | 25.5  [22.7-29.4] | 0.14 |
| **Viral co-detections,** *n* (%) | 112 (26.5) | 150 (21.5) | 24 (19.8) | 44 (29.9) | 17 (27.9) | 3 (11.1) | 109 (25.8) | 0.07 |
| **RSV,** *n* (%) | 36 (8.5) | 25 (3.6)○ | 9 (7.4) | 7 (4.8) | 11 (18.0)○ | 2 (7.4) | 33 (7.8) | 0.008* |
| **Adenovirus (ADV),** *n* (%) | 38 (9.0) | 62 (8.9) | 5 (4.2) | 17 (11.6) | 3 (4.9) | 0 (0.0) | 37 (8.8) | 0.05 |
| **Parainfluenza (PIV),** *n* (%) | 16 (3.8) | 26 (3.7) | 6 (5.0) | 5 (3.4) | 2 (3.3) | 1 (3.7) | 18 (4.3) | 0.99 |
| **hMPV,** *n* (%) | 8 (1.9) | 14 (2.0) | 2 (1.6) | 9 (6.1) | 1 (1.7) | 0 (0.0) | 6 (1.4) | 0.15 |
| **Influenza,** *n* (%) | 2 (0.5) | 1 (0.1) | 0 (0.0) | 0 (0.0) | 0 (0.0) | 0 (0.0) | 3 (0.7) | 0.81 |
| **>1 viral co-detection,** *n* (%) ¶ | 12 (2.8) | 22 (3.2) | 2 (1.6) | 6 (4.0) | 0 (0.0) | 0 (0.0) | 12 (2.8) | 0.64 |
| **Bacterial co-infections** | 9 (2.1)○ | 9 (1.3)○◻△ | 12 (9.9)○ | 2 (1.4)○ | 3 (4.9)◻ | 2 (7.4)○ | 19 (4.5)△ | 0.0001* |
| Blood culture | 0 | 1 | 0 | 0 | 1 | 0 | 2 | 0.28 |
| LRTI/BAL | 9 | 8 | 8 | 2 | 2 | 2 | 17 | 0.65 |
| CF cultures | 0 | 0 | 4** | 0 | 0 | 0 | 0 | ND |

**S4 Table. Viral and bacterial co-infections in children and adolescents with RV infection.**

Continuous variables are expressed as medians 25–75% interquartile ranges (IQR) and categorical data as frequency (%). Analyses by Kruskal-Wallis test with Holm test to Continuous variables are expressed as medians 25–75% interquartile ranges (IQR) and categorical data as frequency (%). Analyses by Kruskal-Wallis test with Holm test to adjust for multiple comparison. Categorical data were analyzed by χ^2^ test. Asterisks (*****) indicate significant *p* values after applying the Bonferroni correction to adjust for multiple testing for families that included multiple parameters. Significant *p* values of <0.05 are represented by blank symbols (○, ◻). Matching symbols indicate the groups for which significant differences were observed. BAL, bronchoalveolar lavage; CF, cystic fibrosis; CHD, congenital heart diseases; hMPV, human metapneumovirus; ICH, immunocompromised host; LRTI, lower respiratory tract infection; RSV, respiratory syncytial virus. **†** Chronic respiratory conditions included: cystic fibrosis (CF) (n=18); chronic lung disease (CLD) (n=61); tracheoesophageal fistula, pulmonary hypertension, spontaneous pneumothorax, airway malacia, subglottic stenosis, cleft palate, and Pierre–Robin sequence. **§** Others included: genetic syndromes (n=132); gastrointestinal diseases (n=68); neurological disorders (n=36); hematologic disease (n=17) and varied including endocrine, osteoarticular or genitourinary underlying diseases (n=169). ¶ Detection of RV and at least two other viruses; RV+2 respiratory viruses in 51 children; RV+3 respiratory viruses in 3 children. ** CF throat cultures from symptomatic children (n=4) were included for a total of four isolates.

**S5 Table. Demographic, viral co-detections and bacterial co-infections in children that died with RV ARI.**

|  | **Condition** | **Age** | **Sex** | **Viral Co-detection** | **RV loads**  **(ct values)** | **Bacterial coinfx*** |
| --- | --- | --- | --- | --- | --- | --- |
| **#1** | **Healthy** | 8 months | Male | **RSV** | 39.5 | ND |
| **#2** | **Healthy** | 6 months | Male | ND | 25.9 | ND |
| **#3** | **Prematurity (34wk)** | 2 months | Male | **RSV** | 21.2 | ND |
| **#4** | **CLD** | 4 months | Female | ND | 21.5 | ***Klebsiella oxytoca*** |
| **#5** | **Endocrine** | 2 years | Male | ND | 16.8 | ND |
| **#6** | **Neurological** | 11 months | Female | ND | 15.1 | ND |
| **#7** | **Asthma/atopy** | 11 years | Male | ND | 31.1 | ND |
| **#8** | **Asthma/atopy** | 7 years | Male | ND | 19.8 | ND |
| **#9** | **Asthma/atopy** | 5 years | Male | ND | 21.1 | ***S. pneumoniae*** |
| **#10** | **Asthma/atopy** | 6 years | Male | **PIV** | 18.5 | ND |
| **#11** | **Asthma/atopy** | 2 years | Male | **hMPV** | 30.6 | ND |
| **#12** | **Asthma/atopy** | 20 years | Female | ND | 36.1 | ND |
| **#13** | **Asthma/atopy** | 15 months | Male | ND | 26.8 | ND |
| **#14** | **CHD** | 23 months | Male | ND | 26.3 | ND |
| **#15** | **CHD** | 3 years | Female | ND | 21.7 | ND |
| **#16** | **ICH** | 20 years | Male | ND | 24.7 | ***Pseudomonas*** |
| **#17** | **ICH** | 9.7 years | Female | ND | 22.1 | ***Scedosporium profilicans*** |
| **#18** | **ICH** | 4 years | Female | ND | 20.5 | ND |
| **#19** | **ICH** | 2 years | Male | ND | 19.5 | ND |
| **#20** | **Genetic syndrome** | 1.5 months | Female | ND | 26.6 | ***Pseudomonas*** |
| **#21** | **Genetic syndrome** | 1.7 months | Female | **ADV** | 25.1 | ND |
| **#22** | **Genetic syndrome** | 1.8 months | Female | ND | 17.1 | ***Klebsiella*** |
| **#23** | **Genetic syndrome** | 2.7 months | Female | ND | 24.3 | ND |
| **#24** | **Genetic syndrome** | 9.3 months | Male | ND | 26.0 | ND |
| **#25** | **Genetic syndrome** | 12.6 months | Female | ND | 23.9 | ND |
| **#26** | **Genetic syndrome** | 13 months | Male | ND | 37.3 | ND |
| **#27** | **Genetic syndrome** | 4.8 months | Male | ND | 21.7 | ND |
| **#29** | **Genetic syndrome** | 5.3 months | Male | ND | 23.2 | ND |
| **#29** | **Genetic syndrome** | 15 years | Female | **ADV** | 23.1 | ND |
| **#30** | **Genetic syndrome** | 18 years | Male | ND | 36.6 | ND |

*All bacterial pathogens identified were cultured from the lower respiratory tract. ADV, adenovirus; CHD, congenital heart diseases; CLD, chronic lung disease; hMPV, human metapneumovirus; ICH, immunocompromised host; PIV, parainfluenza virus; RSV, respiratory syncytial virus; ND, not detected.
