# Supplementary material for: The differential burden of acute rhinovirus infections in children with underlying conditions
Source: PLoS One. 2025 May 14;20(5):e0313237. doi: 10.1371/journal.pone.0313237 (PMC12077780; doi:10.1371/journal.pone.0313237)
Supplement: Attachment — Submitted filename: Response to reviewers_R2.docx [file pone.0313237.s004.docx]

**PONE-D-24-47497R1**

*The differential burden of acute rhinovirus infections in children with underlying conditions*

PLOS ONE

Dear Editor, as requested we have included the following items in the revised version of the manuscript:

- A rebuttal letter that responds to each point raised by the academic editor and reviewer(s). You should upload this letter as a separate file labeled 'Response to Reviewers'.

***It appears that the last resubmission (R1) satisfied all previous comments and concerns raised by the reviewers and editors. There were no specific comments for us to address in the manuscript version that we received last week. Please see below Reviewer #1 comments.***

- A marked-up copy of your manuscript that highlights changes made to the original version. You should upload this as a separate file labeled 'Revised Manuscript with Track Changes'.

***There were only changes requested regarding the references, which we have updated. These references are now highlighted in yellow.***

- An unmarked version of your revised paper without tracked changes. You should upload this as a separate file labeled 'Manuscript'.

***This has been included***

If you would like to make changes to your financial disclosure, please include your updated statement in your cover letter. Guidelines for resubmitting your figure files are available below the reviewer comments at the end of this letter.

**No changes have occurred in our COI**

We look forward to receiving your revised manuscript.

Kind regards,

Tai-Heng Chen, M.D., Ph.D.
Academic Editor
PLOS ONE

**Journal Requirements**:
Please review your reference list to ensure that it is complete and correct. If you have cited papers that have been retracted, please include the rationale for doing so in the manuscript text or remove these references and replace them with relevant current references. Any changes to the reference list should be mentioned in the rebuttal letter that accompanies your revised manuscript. If you need to cite a retracted article, indicate the article’s retracted status in the References list and also include a citation and full reference for the retraction notice.

Response:

**We have thoroughly reviewed our reference list and have made minor changes that are highlighted in the revised version of the manuscript. Specifically:**

**Introduction:**

- Reference #6 which was previously located in page 3, line 6 has been moved to the previous line (line 5, page 3), as it was originally misplaced.

**Discussion**

- Page 13, line 1: We have updated the three previous references (10, 28 and 29) and are now #8, 11 and 18. The three updated references reflect better the content of the discussion.
- Page 13, lines 4 and 5: we have added three new references, 28, 29 and 31 and mention a previously cited reference (#30) that was relevant to the topic discussed in that section.
- Page 13, line 8, reference 33 has been moved to this section that fits better with the content of the sentence.

**Reviewers' comments:**
Reviewer's Responses to Questions

***Reviewers did not have specific questions or comments for us to address. We thank them for their previous comments and suggestions (R1) that had greatly improved our manuscript.***

**Comments to the Author**
1. If the authors have adequately addressed your comments raised in a previous round of review and you feel that this manuscript is now acceptable for publication, you may indicate that here to bypass the “Comments to the Author” section, enter your conflict of interest statement in the “Confidential to Editor” section, and submit your "Accept" recommendation.

Reviewer #1: All comments have been addressed

2. Is the manuscript technically sound, and do the data support the conclusions?
The manuscript must describe a technically sound piece of scientific research with data that supports the conclusions. Experiments must have been conducted rigorously, with appropriate controls, replication, and sample sizes. The conclusions must be drawn appropriately based on the data presented.

Reviewer #1: Yes

3. Has the statistical analysis been performed appropriately and rigorously?

Reviewer #1: Yes

4. Have the authors made all data underlying the findings in their manuscript fully available?

The [PLOS Data policy](https://nam11.safelinks.protection.outlook.com/?url=http%3A%2F%2Ftrack.editorialmanager.com%2FCL0%2Fhttp%3A%252F%252Fwww.plosone.org%252Fstatic%252Fpolicies.action%2523sharing%2F1%2F010f019535fb5d2c-14db324d-58ad-4bae-8c4e-1b643c5ef81d-000000%2F5GKbGmiweQ2zgM1xQApMxRrqzjLWzd5NuQe1TqWd1sY%3D199&data=05%7C02%7Casuncion.mejias%40stjude.org%7C905d2895852c47ba86b108dd5482a301%7C22340fa892264871b677d3b3e377af72%7C0%7C0%7C638759642264388258%7CUnknown%7CTWFpbGZsb3d8eyJFbXB0eU1hcGkiOnRydWUsIlYiOiIwLjAuMDAwMCIsIlAiOiJXaW4zMiIsIkFOIjoiTWFpbCIsIldUIjoyfQ%3D%3D%7C0%7C%7C%7C&sdata=EM4QEEbIBT4hZSqzqn23v8KWe221oAMerW%2Fe7wvzMV0%3D&reserved=0) requires authors to make all data underlying the findings described in their manuscript fully available without restriction, with rare exception (please refer to the Data Availability Statement in the manuscript PDF file). The data should be provided as part of the manuscript or its supporting information, or deposited to a public repository. For example, in addition to summary statistics, the data points behind means, medians and variance measures should be available. If there are restrictions on publicly sharing data—e.g. participant privacy or use of data from a third party—those must be specified.

Reviewer #1: Yes

5. Is the manuscript presented in an intelligible fashion and written in standard English?

PLOS ONE does not copyedit accepted manuscripts, so the language in submitted articles must be clear, correct, and unambiguous. Any typographical or grammatical errors should be corrected at revision, so please note any specific errors here.

Reviewer #1: Yes

6. Review Comments to the Author

Please use the space provided to explain your answers to the questions above. You may also include additional comments for the author, including concerns about dual publication, research ethics, or publication ethics. (Please upload your review as an attachment if it exceeds 20,000 characters)

Reviewer #1: (No Response)

7. PLOS authors have the option to publish the peer review history of their article ([what does this mean?](https://nam11.safelinks.protection.outlook.com/?url=https%3A%2F%2Ftrack.editorialmanager.com%2FCL0%2Fhttps%3A%252F%252Fjournals.plos.org%252Fplosone%252Fs%252Feditorial-and-peer-review-process%2523loc-peer-review-history%2F1%2F010f019535fb5d2c-14db324d-58ad-4bae-8c4e-1b643c5ef81d-000000%2FRp-yICmRWnwdfJWQ682xZ0SVqGIxTchkeRVseGVaVSo%3D199&data=05%7C02%7Casuncion.mejias%40stjude.org%7C905d2895852c47ba86b108dd5482a301%7C22340fa892264871b677d3b3e377af72%7C0%7C0%7C638759642264403228%7CUnknown%7CTWFpbGZsb3d8eyJFbXB0eU1hcGkiOnRydWUsIlYiOiIwLjAuMDAwMCIsIlAiOiJXaW4zMiIsIkFOIjoiTWFpbCIsIldUIjoyfQ%3D%3D%7C0%7C%7C%7C&sdata=ow309gGkOJYyyuh2GDhdBUwKhsSq7%2FuO%2BxC%2Bq51%2BDE8%3D&reserved=0)). If published, this will include your full peer review and any attached files.
If you choose “no”, your identity will remain anonymous but your review may still be made public.

**Do you want your identity to be public for this peer review?** For information about this choice, including consent withdrawal, please see our [Privacy Policy](https://nam11.safelinks.protection.outlook.com/?url=https%3A%2F%2Ftrack.editorialmanager.com%2FCL0%2Fhttps%3A%252F%252Fwww.plos.org%252Fprivacy-policy%2F1%2F010f019535fb5d2c-14db324d-58ad-4bae-8c4e-1b643c5ef81d-000000%2FQBkjWl5AyJVHJWWibOLiBw-z4DIhpkSCp2GxP3pF6i8%3D199&data=05%7C02%7Casuncion.mejias%40stjude.org%7C905d2895852c47ba86b108dd5482a301%7C22340fa892264871b677d3b3e377af72%7C0%7C0%7C638759642264417968%7CUnknown%7CTWFpbGZsb3d8eyJFbXB0eU1hcGkiOnRydWUsIlYiOiIwLjAuMDAwMCIsIlAiOiJXaW4zMiIsIkFOIjoiTWFpbCIsIldUIjoyfQ%3D%3D%7C0%7C%7C%7C&sdata=U97oVa7ZH0RrkzTj%2B6EF4iIwBt3gsMY6QZ1R6r9OMWQ%3D&reserved=0).

Reviewer #1: No
